# Structural Basis for pH-Dependent Oligomerization of Dihydropyrimidinase from *Pseudomonas aeruginosa* PAO1

**DOI:** 10.1155/2018/9564391

**Published:** 2018-01-30

**Authors:** Jen-Hao Cheng, Chien-Chih Huang, Yen-Hua Huang, Cheng-Yang Huang

**Affiliations:** ^1^School of Biomedical Sciences, Chung Shan Medical University, No. 110, Sec. 1, Chien-Kuo N. Rd., Taichung, Taiwan; ^2^Department of Medical Research, Chung Shan Medical University Hospital, No. 110, Sec. 1, Chien-Kuo N. Rd., Taichung, Taiwan

## Abstract

Dihydropyrimidinase, a dimetalloenzyme containing a carboxylated lysine within the active site, is a member of the cyclic amidohydrolase family, which also includes allantoinase, dihydroorotase, hydantoinase, and imidase. Unlike all known dihydropyrimidinases, which are tetrameric, pseudomonal dihydropyrimidinase forms a dimer at neutral pH. In this paper, we report the crystal structure of *P. aeruginosa* dihydropyrimidinase at pH 5.9 (PDB entry 5YKD). The crystals of *P. aeruginosa* dihydropyrimidinase belonged to space group *C*222_1_ with cell dimensions of *a* = 108.9, *b* = 155.7, and *c* = 235.6 Å. The structure of *P. aeruginosa* dihydropyrimidinase was solved at 2.17 Å resolution. An asymmetric unit of the crystal contained four crystallographically independent *P. aeruginosa* dihydropyrimidinase monomers. Gel filtration chromatographic analysis of purified *P. aeruginosa* dihydropyrimidinase revealed a mixture of dimers and tetramers at pH 5.9. Thus, *P. aeruginosa* dihydropyrimidinase can form a stable tetramer both in the crystalline state and in the solution. Based on sequence analysis and structural comparison of the dimer-dimer interface between *P. aeruginosa* dihydropyrimidinase and *Thermus* sp. dihydropyrimidinase, different oligomerization mechanisms are proposed.

## 1. Introduction

**Figure 1:**
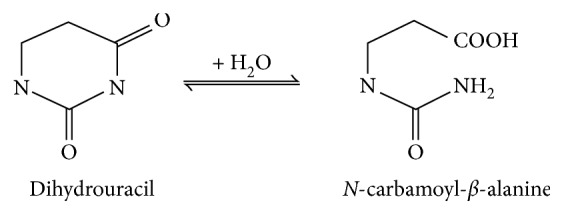
The physiological reaction of dihydropyrimidinase. Dihydropyrimidinase catalyzes the reversible cyclization of dihydrouracil to *N*-carbamoyl-*β*-alanine in the second step of the pyrimidine degradation pathway.

Dihydropyrimidinase is a key enzyme for pyrimidine catabolism [1, 2]. Dihydropyrimidinase catalyzes the reversible cyclization of dihydrouracil to *N*-carbamoyl-*β*-alanine in the second step of the pyrimidine degradation pathway (Figure 1). Dihydropyrimidinase can also detoxify xenobiotics with an imide functional group, ranging from linear imides to heterocyclic imides [3–9]. Homologous enzymes from microorganisms are known as hydantoinase, used as biocatalyst for hydrolysis of 5-monosubstituted hydantoins in the synthesis of D- and L-amino acids [10, 11]. Optically pure amino acids have been widely used as intermediates for semisynthesis of antibiotics, active peptides, hormones, antifungal agents, pesticides, and sweeteners. Dihydropyrimidinase and hydantoinase generally possess a similar active site, but their overall sequence identity and substrate specificity may differ [3, 12]. For example, hydantoinase purified from *Agrobacterium* species has no 5,6-dihydropyrimidine amidohydrolase activity [13]. Dihydropyrimidinases from the yeast *Saccharomyces kluyveri* and the slime mold *Dictyostelium discoideum* do not hydrolyze hydantoin [14]. Thus, several bacterial hydantoinases are still named and identified as dihydropyrimidinase because of their catalytic activity toward natural substrates, namely, dihydrouracil and dihydrothymine. These bacterial enzymes include *Pseudomonas aeruginosa* and *Thermus* sp. dihydropyrimidinases [15, 16].

Dihydropyrimidinase, hydantoinase, imidase, allantoinase, and dihydroorotase belong to the cyclic amidohydrolase family because of their functional and structural similarities [17]. Members of this enzyme family catalyze the ring-opening hydrolysis of the cyclic amide bond of each substrate in either five- or six-membered rings. Even if these enzymes have similar functions, they have relatively low amino acid sequence identity. In addition, the substrate selectivity and specificity of these enzymes highly differ [18, 19]. Most of the active sites of dihydropyrimidinases, hydantoinases, allantoinases, and dihydroorotases contain four histidines, one aspartate, and one carboxylated lysine residue, which are required for metal binding and catalytic activity [8, 15, 18, 20, 21]. The presence of a carboxylated lysine in hydantoinase is also required for the self-assembly of the binuclear metal center [12, 20, 22] and increases the nucleophilicity of the hydroxide for catalysis [23]. The global architecture of the dihydropyrimidinase monomer consists of two domains, namely, a large domain with a classic (*β*/*α*)_8_-barrel structure core embedding the catalytic dimetal center and a small *β*-sandwich domain [16, 22, 24, 25].

All known dihydropyrimidinases are tetramers except pseudomonal enzymes. Hydantoinase from *P*. *putida* YZ-26 functions as a dimer [26, 27]. Recently, we identified that dihydropyrimidinase from *P. aeruginosa* PAO1 also forms a dimer [28]. In addition, the crystal structure of *P. aeruginosa* PAO1 dihydropyrimidinase indicated that several residues crucial for tetramerization are not found in *P. aeruginosa* dihydropyrimidinase [28]. In this study, we found that the oligomerization of *P. aeruginosa* PAO1 dihydropyrimidinase is a pH-dependent process. At pH 5.9, *P. aeruginosa* PAO1 dihydropyrimidinase mainly formed a tetramer. To confirm this result and determine how this enzyme can also form a tetramer, we also determined the crystal structure of *P. aeruginosa* PAO1 dihydropyrimidinase at 2.17 Å resolution at acidic environment. Structural comparison indicated that although *P. aeruginosa* PAO1 dihydropyrimidinase can also form a tetramer, the residues being crucial for tetramerization are different from those in *Thermus* sp. dihydropyrimidinases.

## 2. Materials and Methods

### 2.1. Cloning, Protein Expression, and Purification

Construction of the *P. aeruginosa* dihydropyrimidinase expression plasmid has been reported [15]. Recombinant *P. aeruginosa* dihydropyrimidinase was expressed and purified using the protocol described previously [15]. The protein purified from the soluble supernatant by Ni^2+^-affinity chromatography (HiTrap HP; GE Healthcare Bio-Sciences, Piscataway, NJ, USA) was eluted with Buffer A (20 mM Tris-HCl, 250 mM imidazole, and 0.5 M NaCl, pH 7.9) and dialyzed against a dialysis buffer (20 mM HEPES and 100 mM NaCl, pH 7.0; Buffer B). Protein purity remained > 97% as determined by SDS-PAGE (Mini-PROTEAN Tetra System; Bio-Rad, CA, USA).

### 2.2. Gel Filtration Chromatography

Gel filtration chromatography was carried out by the AKTA-FPLC system (GE Healthcare Bio-Sciences, Piscataway, NJ, USA). In brief, purified protein (5 mg/mL) in Buffer C (20 mM MES and 100 mM NaCl, pH 5.9) was applied to a Superdex 200 prep grade column (GE Healthcare Bio-Sciences, Piscataway, NJ, USA) equilibrated with the same buffer [29]. The column was operated at a flow rate of 0.5 mL/min, and the proteins were detected at 280 nm. The column was calibrated with proteins of known molecular weight: thyroglobulin (670 kDa), *γ*-globulin (158 kDa), ovalbumin (44 kDa), myoglobin (17 kDa), and vitamin B_12_ (1.35 kDa).

### 2.3. Crystallography

Before crystallization, *P. aeruginosa* dihydropyrimidinase was concentrated to 20 mg/mL in Buffer C. Crystals were grown at room temperature by hanging drop vapor diffusion in 10% PEG 8000, 100 mM HEPES, 200 mM calcium acetate, pH 5.9. Data collection and refinement statistics for the crystal of *P. aeruginosa* dihydropyrimidinase are shown in Table 1. Data were collected using an ADSC Quantum-315r CCD area detector at SPXF beamline BL13C1 at NSRRC (Taiwan, ROC). All data integration and scaling were carried out using HKL-2000 [30]. There were four *P. aeruginosa* dihydropyrimidinase monomers per asymmetric unit. The crystal structure of *P. aeruginosa* dihydropyrimidinase was solved at 2.17 Å resolution with the molecular replacement software AMoRe [31] using the dihydropyrimidinase (PDB entry 5E5C) [28] as model. After molecular replacement, model building was carried out using XtalView [32]. CNS was used for molecular dynamics refinement [33]. The final structure was refined to an *R*-factor of 0.1759 and an *R*_free_ of 0.2312. Atomic coordinates and related structural factors have been deposited in the PDB with accession code 5YKD.

## 3. Results and Discussion

### 3.1. Structure of the *P. aeruginosa* Dihydropyrimidinase Monomer

**Figure 2:**
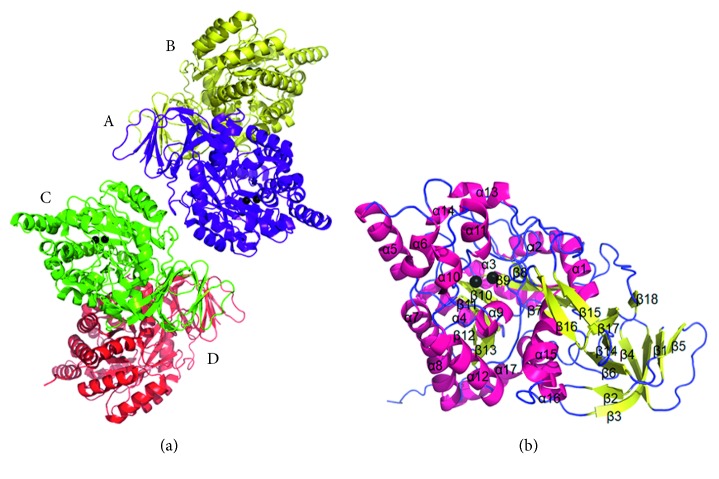
Crystal structure of *P. aeruginosa* dihydropyrimidinase. (a) Ribbon diagram of a *P. aeruginosa* dihydropyrimidinase tetramer. Each *P. aeruginosa* dihydropyrimidinase monomer is color-coded. Two zinc ions in the active site are presented as black spheres. (b) Ribbon diagram of a *P. aeruginosa* dihydropyrimidinase monomer with the secondary structures labeled.

Crystals of *P. aeruginosa* dihydropyrimidinase were grown at room temperature by hanging drop vapor diffusion in 10% PEG 8000, 100 mM HEPES, 200 mM calcium acetate, pH 5.9. The crystals of *P. aeruginosa* dihydropyrimidinase grown under this condition belonged to space group *C*222_1_ with cell dimensions of *a* = 108.9, *b* = 155.7, and *c* = 235.6 Å. The crystal structure of *P. aeruginosa* dihydropyrimidinase was solved at 2.17 Å resolution (Table 1). The unit cell contained eight molecules. An asymmetric unit of the crystal contained four crystallographically independent *P. aeruginosa* dihydropyrimidinase monomers, in which two zinc ions were found in the active site per monomer (Figure 2). The majority of the electron density for *P. aeruginosa* dihydropyrimidinase exhibited good quality, and no discontinuity was observed. Briefly, the overall structure of each *P. aeruginosa* dihydropyrimidinase unit consists of 17 *α*-helices, 19 *β*-sheets, and two zinc ions (Figure 2). At pH 5.9, the architecture of the *P. aeruginosa* dihydropyrimidinase monomer consists of two domains, namely, a large domain with a classic (*β*/*α*)_8_-barrel structure core embedding the catalytic dimetal center and a small *β*-sandwich domain.

### 3.2. Structural Comparison

**Figure 3:**
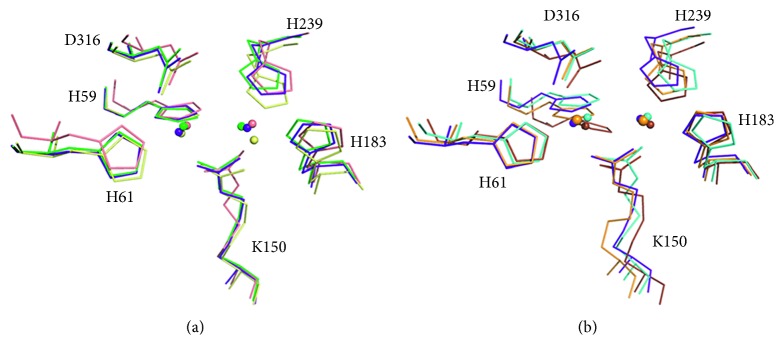
Structural comparison. (a) Superposition of the active site of dihydropyrimidinases. Their active sites contain four histidines, one aspartate, and one carboxylated lysine residue, which are required for metal binding and catalytic activity. Dihydropyrimidinases from *P. aeruginosa* (PDB entry 5E5C; green), *Thermus* sp. (PDB entry 1GKQ; salmon), *Tetraodon nigroviridis* (PDB entry 4H01; pale yellow), and the structure (PDB entry 5YKD; purple blue) in this study are shown. The architecture of these active sites is similar. (b) Superposition of the active site of members of the amidohydrolase family. Their active sites contain four histidines, one aspartate, and one carboxylated lysine residue, which are required for metal binding and catalytic activity. *P. aeruginosa* dihydropyrimidinase (PDB entry 5YKD; purple blue), *Escherichia coli* allantoinase (PDB entry 3E74; bright orange), *Burkholderia pickettii* hydantoinase (PDB entry 1NFG; aquamarine), and *E. coli* dihydroorotase (PDB entry 1J79; brown) are shown. The architecture of these active sites is similar.

The overall structure and architecture of the active site of *P. aeruginosa* dihydropyrimidinase are similar to those of other dihydropyrimidinases (Figure 3) and other members of the amidohydrolase family of enzymes, such as hydantoinases, dihydroorotases, and allantoinases (Figure 3). The active sites of these enzymes contain four histidines, one aspartate, and one carboxylated lysine residue, which are required for metal binding and catalytic activity [12, 14, 15, 19, 20, 34, 35].

### 3.3. pH-Dependent Oligomerization of *P. aeruginosa* Dihydropyrimidinase

**Figure 4:**
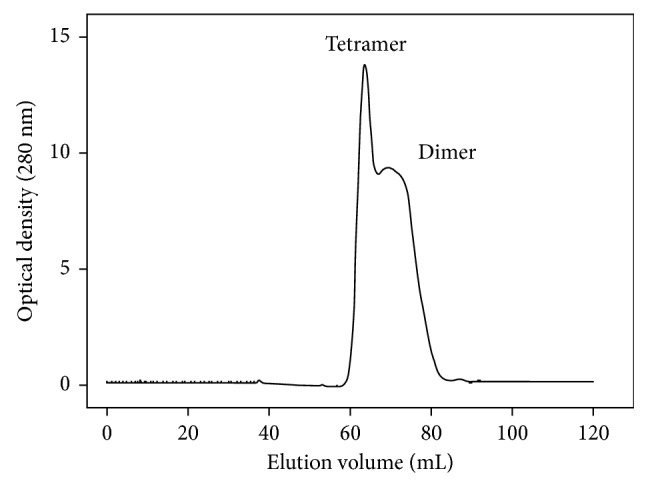
Gel filtration chromatographic analysis. Gel filtration chromatography was carried out by the AKTA-FPLC system in Buffer C (20 mM MES and 100 mM NaCl, pH 5.9). The corresponding peaks show the eluting *P. aeruginosa* dihydropyrimidinase. The column was calibrated with proteins of known molecular weight: thyroglobulin (670 kDa), *γ*-globulin (158 kDa), ovalbumin (44 kDa), myoglobin (17 kDa), and vitamin B_12_ (1.35 kDa).

It was noted that the crystals of the dimeric *P. aeruginosa* dihydropyrimidinase belonged to space group *P*3_1_21 grown at the condition of 28% PEG 6000, 100 mM HEPES, 200 mM lithium acetate, pH 7.5 [28]. Due to the different crystallization condition, we attempted to test whether the oligomerization of *P. aeruginosa* dihydropyrimidinase is pH-dependent. All known dihydropyrimidinases are tetramers. However, pseudomonal dihydropyrimidinase/hydantoinase forms a dimer at neutral pH [26–28]. Given that the structure implies that *P. aeruginosa* dihydropyrimidinase may also form a tetramer in the crystalline state at pH 5.9 (Figure 2), we performed biochemical verification to confirm the oligomerization state. To confirm whether or not the oligomerization of *P. aeruginosa* dihydropyrimidinase is pH-dependent, we conducted gel filtration chromatography at pH 5.9. As shown in Figure 4, the results revealed that two species with elution volume of 63.25 and 69. 26 mL did coexist. The molecular mass of a *P. aeruginosa* dihydropyrimidinase monomer, as calculated from the amino acid sequence, is 53 kDa. Assuming that these two forms of *P. aeruginosa* dihydropyrimidinase have a shape and partial specific volume similar to the standard proteins, the native molecular masses of *P. aeruginosa* dihydropyrimidinase were estimated to be 105 and 180 kDa, approximately 1.9 and 3.5 times the molecular mass of a *P. aeruginosa* dihydropyrimidinase monomer, respectively. In comparison at pH 7.5, gel filtration chromatographic analysis of *P. aeruginosa* dihydropyrimidinase revealed a single peak; the native molecular mass was estimated to be 117 kDa [28]. The two forms of this enzyme obtained from the gel filtration chromatography at pH 5.9 had similar specific activity (data not shown). Thus, *P. aeruginosa* dihydropyrimidinase did exist as a mixture of dimers and tetramers at pH 5.9.

### 3.4. Structural Insights into Dimer of Dimer (Tetramer) Formation of Dihydropyrimidinase

**Figure 5:**
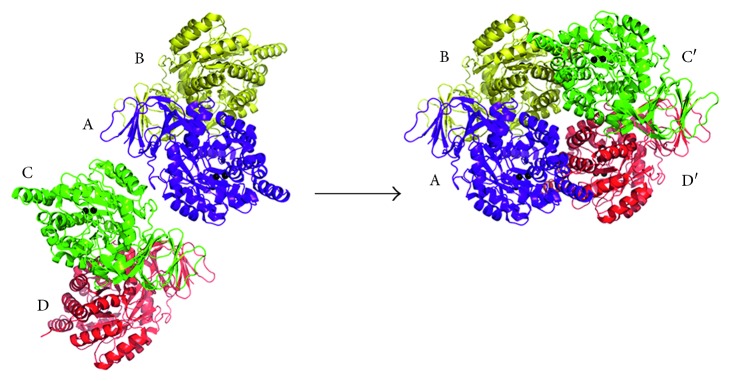
The structure of *P. aeruginosa* dihydropyrimidinase tetramer. An asymmetric unit contains four crystallographically independent *P. aeruginosa* dihydropyrimidinase monomers B-A-C-D. Crystallographically related tetramer B-A-C′-D′ was formed and further stabilized via many hydrogen bonds and salt bridges. This tetramerization structure was similar to that of *Thermus* sp. dihydropyrimidinase (PDB entry 1GKQ).

In this study, we have identified that *P. aeruginosa* dihydropyrimidinase did exist as a mixture of dimers and tetramers at pH 5.9. To assess how *P. aeruginosa* dihydropyrimidinase can form a stable tetramer, the dimer-dimer interface was analyzed. In the crystal of *P. aeruginosa* dihydropyrimidinase, the four molecules formed two pairs of dimers, B-A and C-D, respectively (Figure 5). Since the two dimers of *P. aeruginosa* dihydropyrimidinase associate via few contacts to create the tetramer, it was thought that the tetrameric state may be possibly due to crystal packing forces. We noted that in the crystal, another crystallographically related tetramer B-A-C′-D′ (Figure 5) was formed and further stabilized via many hydrogen bonds and salt bridges (Tables 2 and 3). This tetramerization structure was similar to that of *Thermus* sp. dihydropyrimidinase (PDB entry 1GKQ).

**Figure 6:**
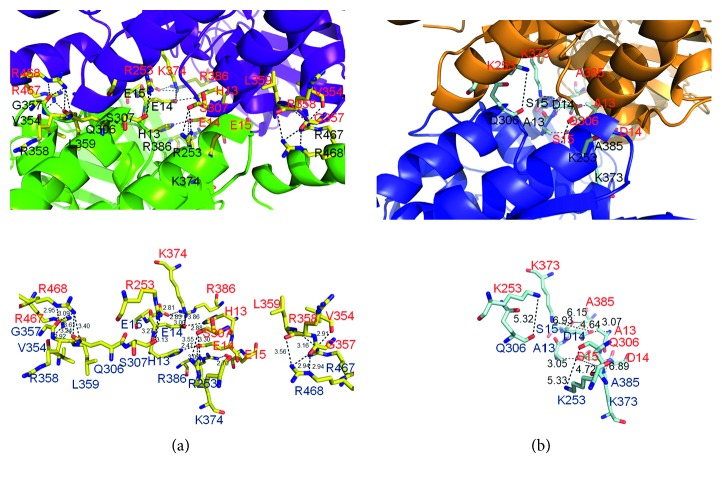
Comparison of the tetrameric structures of *Thermus* sp. dihydropyrimidinase and *P. aeruginosa* dihydropyrimidinase. (a) Structural analysis of the dimer-dimer interface of *P. aeruginosa* dihydropyrimidinase. The distance (Å) of the residues is shown. (b) Many residues crucial for forming hydrogen bonds at the dimer-dimer interface of *P. aeruginosa* dihydropyrimidinase were not found in the dimer-dimer interface of *Thermus* sp. dihydropyrimidinase.

**Figure 7:**
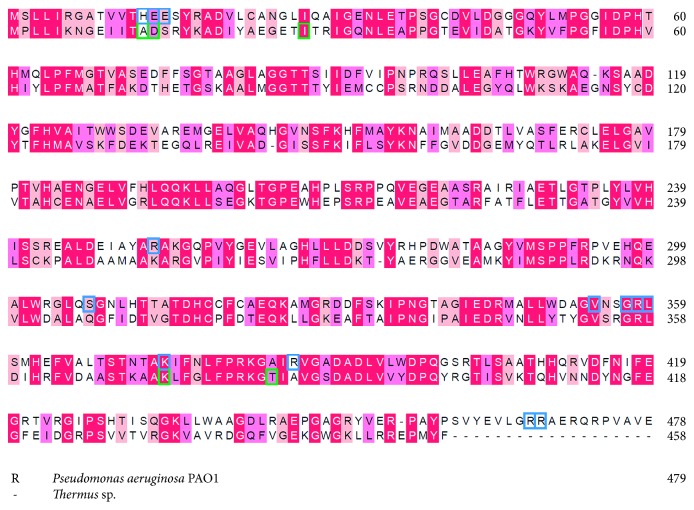
Sequence alignment of dihydropyrimidinases from *P. aeruginosa* and *Thermus* sp. The amino acids that are involved in dimer-dimer interface of *P. aeruginosa* and *Thermus* sp. dihydropyrimidinase are boxed, respectively.

We also compared the residues important for tetramerization located at the B-A-C′-D′ dimer-dimer interface with those of *Thermus* sp. dihydropyrimidinase (Figure 6). Although their overall structures are similar, the important residues for tetramer (dimer B-C′ with dimer A-D′) formation are quite different. For the tetramer formation of *P. aeruginosa* dihydropyrimidinase, many hydrogen bonds with close distance were found: these bonds (<3 Å) include K374(A)–E14(B), H13(A)–E14(B), R386(A)–E14(B), R386(A)–E15(B), R467(A)–V354(B), R468(A)–G357(B), E14(A)–H13(B), E15(A)–R386(B), V354(A)–R467(B), G357(A)–R468(B), H13(C′)–E14(D′), R386(C′)–E15(D′), R468(C′)–G357(D′), E14(C′)–H13(D′), E14(C′)–K374(D′), E15(C′)–R386(D′), and G357(C′)–R468(D′); however, these residues were not found for the tetramer formation of *Thermus* sp. dihydropyrimidinase (Figure 6). Only A13–D14 hydrogen bond was found in *Thermus* sp. dihydropyrimidinase (i.e., H13–E14 in *P. aeruginosa* dihydropyrimidinase). Thus, the dimer-dimer interface between *P. aeruginosa* dihydropyrimidinase and *Thermus* sp. dihydropyrimidinase was significantly different (Figure 7). Comparison by superimposition indicated that many Arg residues (R253, R358, R386, R467, and R468) found in *P. aeruginosa* dihydropyrimidinase, but not in *Thermus* sp. dihydropyrimidinase, may play a crucial role for the pH-dependent oligomerization. If consider the p*K*_a_, a much better candidate is His13, which is involved in intermolecular interactions and, dependent on the environment of its side chain, which may easily change protonation state between pH 5.9 and pH 7.5. However, this speculation needs to be confirmed by further biochemical experiments.

### 3.5. Different Mechanisms for Tetramer Formation of Dihydropyrimidinases

In this study, we identified *P. aeruginosa* dihydropyrimidinase can be a tetramer both in the crystalline state and in solution (Figure 4). The structure of the tetrameric *Thermus* sp. dihydropyrimidinase and *P. aeruginosa* dihydropyrimidinase was compared (Figure 6). Many important residues for *Thermus* sp. dihydropyrimidinase tetramer formation are different from those for *P. aeruginosa* dihydropyrimidinase (Figure 7). On the basis of these results, we concluded that *P. aeruginosa* dihydropyrimidinase could form a tetramer, but its oligomerization mechanism differed from those of other dihydropyrimidinases such as *Thermus* sp. dihydropyrimidinase.

## Figures and Tables

**Table 1:** Data collection and refinement statistics.

Data collection	
Crystal	*P. aeruginosa* dihydropyrimidinase
Wavelength (Å)	0.975
Resolution (Å)	30–2.17
Space group	*C*222_1_
Cell dimension (Å)	*a* = 108.9, *α* = 90
	*b* = 155.7, *β* = 90
	*c* = 235.6, *γ* = 120
Completeness (%)	99.8 (100)^∗^
<*I*/*σI*>	15.13 (3.7)
*R*_sym_ or *R*_merge_ (%)	0.122 (0.599)
Redundancy	7.1 (7.3)
Refinement	
Resolution (Å)	30–2.17
Number of reflections	100197
*R*_work_/*R*_free_	0.1759/0.2312
Number of atoms	
Protein	1912
Water	312
RMS deviation	
Bond lengths (Å)	0.0151
Bond angles (°)	1.6495
Ramachandran plot	
In preferred regions	1345 (94.19%)
In allowed regions	68 (4.76%)
Outliers	15 (1.05%)
PDB entry	5YKD

^∗^Values in parentheses are for the highest resolution shell.

**Table 2:** The formation of hydrogen bonds at the dimer-dimer interface of *P. aeruginosa* dihydropyrimidinase.

Subunit 1	Distance [Å]	Subunit 2
A: K374 [NZ]	3.00	B: E14 [OE1]
A: H13 [NE2]	2.88	B: E14 [OE1]
A: R386 [NH2]	3.86	B: E14 [OE2]
A: R386 [NH1]	2.81	B: E15 [OE2]
A: R386 [NH2]	2.83	B: E15 [OE2]
A: R468 [NH2]	3.61	B: Q306 [OE1]
A: R253 [NH1]	3.27	B: S307 [O]
A: R253 [NH2]	3.13	B: S307 [O]
A: R467 [NH1]	2.92	B: V354 [O]
A: R468 [NE]	2.95	B: G357 [O]
A: R468 [NH2]	3.09	B: G357 [O]
A: R468 [NH2]	3.40	B: R358 [O]
A: R467 [NH1]	3.24	B: L359 [O]
A: E14 [OE1]	3.09	B: K374 [NZ]
A: E14 [OE1]	2.47	B: H13 [NE2]
A: E15 [OE2]	2.70	B: R386 [NH1]
A: S307 [O]	3.30	B: R253 [NH1]
A: S307 [O]	3.55	B: R253 [NH2]
A: V354 [O]	2.91	B: R467 [NH1]
A: G357 [O]	2.94	B: R468 [NH2]
A: G357 [O]	2.94	B: R468 [NE]
A: R358 [O]	3.56	B: R468 [NH2]
A: L359 [O]	3.16	B: R467 [NH1]
C′: H13 [NE2]	2.79	D′: E14 [OE1]
C′: K374 [NZ]	3.25	D′: E14 [OE1]
C′: R386 [NH1]	2.85	D′: E15 [OE1]
C′: R386 [NH2]	2.59	D′: E15 [OE2]
C′: R468 [NH2]	3.26	D′: Q306 [OE1]
C′: R253 [NH1]	3.13	D′: S307 [O]
C′: R253 [NH2]	3.16	D′: S307 [O]
C′: R468 [NE]	2.71	D′: G357 [O]
C′: R468 [NH2]	3.11	D′: R358 [O]
C′: E14 [OE1]	2.88	D′: H13 [NE2]
C′: E14 [OE1]	2.89	D′: K374 [NZ]
C′: E15 [OE2]	2.88	D′: R386 [NH1]
C′: E15 [OE2]	2.73	D′: R386 [NH2]
C′: Q306 [OE1]	3.53	D′: R468 [NH2]
C′: S307 [O]	3.21	D′: R253 [NH1]
C′: S307 [O]	3.59	D′: R253 [NH2]
C′: G357 [O]	2.65	D′: R468 [NE]
C′: R358 [O]	3.33	D′: R468 [NH2]

**Table 3:** The formation of salt bridges at the dimer-dimer interface of *P. aeruginosa* dihydropyrimidinase.

Subunit 1	Distance [Å]	Subunit 2
A: K374 [NZ]	3.00	B: E14 [OE1]
A: H13 [NE2]	2.88	B: E14 [OE1]
A: R386 [NH2]	3.86	B: E14 [OE2]
A: H13 [NE2]	3.75	B: E14 [OE2]
A: R386 [NH1]	3.55	B: E15 [OE1]
A: R386 [NH1]	2.81	B: E15 [OE2]
A: R386 [NH2]	2.83	B: E15 [OE2]
A: E14 [OE1]	3.09	B: K374 [NZ]
A: E14 [OE1]	2.47	B: H13 [NE2]
A: E14 [OE2]	3.93	B: H13 [NE2]
A: E15 [OE1]	3.69	B: R386 [NH1]
A: E15 [OE2]	3.00	B: R386 [NH2]
A: E15 [OE2]	2.70	B: R386 [NH1]
C′: H13 [NE2]	2.79	D′: E14 [OE1]
C′: K374 [NZ]	3.25	D′: E14 [OE1]
C′: H13 [NE2]	3.86	D′: E14 [OE2]
C′: R386 [NH1]	2.85	D′: E15 [OE1]
C′: R386 [NH2]	3.84	D′: E15 [OE1]
C′: R386 [NH1]	2.96	D′: E15 [OE2]
C′: R386 [NH2]	2.59	D′: E15 [OE2]
C′: E14 [OE1]	2.88	D′: H13 [NE2]
C′: E14 [OE1]	2.89	D′: K374 [NZ]
C′: E14 [OE2]	3.78	D′: H13 [NE2]
C′: E15 [OE1]	3.34	D′: R386 [NH1]
C′: E15 [OE2]	2.88	D′: R386 [NH1]
C′: E15 [OE2]	2.73	D′: R386 [NH2]
